# A Case Report of Laparoscopic-Assisted Repair of a Rare Congenital Lumbar (Grynfeltt-Lesshaft) Hernia

**DOI:** 10.7759/cureus.73239

**Published:** 2024-11-07

**Authors:** Panuwat Pornkul, Helen B Buschel, Daniel Carroll

**Affiliations:** 1 Division of General Surgery, Townsville Hospital and Health Service, Townsville, AUS; 2 Department of Surgery, School of Medicine, James Cook University, Townsville, AUS; 3 Division of Paediatric Surgery, Department of Health and Well-Being, Townsville Hospital and Health Service, Townsville, AUS

**Keywords:** case report, congenital lumbar hernia, flank hernia, laparoscopic surgery, laparoscopy, lumbar hernia

## Abstract

A congenital lumbar hernia is a rare type of hernia that can affect children born with lumbo-costo-vertebral syndrome. This case report is the first to describe a hybrid laparoscopic-assisted approach, which enabled precise intra-operative localization of a pediatric congenital lumbar hernia, and definitive surgical repair was then undertaken through an open approach. Unlike prior studies that have focused solely on either open or laparoscopic techniques, this hybrid approach offers a new strategy to improve surgical accuracy, particularly where imaging and clinical examination are inconclusive. Our literature review found that open and laparoscopic repair of congenital lumbar hernias are both safe and feasible approaches, with the choice of either depending on the surgeon's preference and expertise.

## Introduction

Congenital lumbar hernia is a rare entity, and as a result, there is a paucity of literature on the pediatric population. Surgical repair is the primary treatment, with most documented cases using an open approach. More recently, there has been a small number of case reports describing a laparoscopic approach [1,2]. This case report describes successful laparoscopic-assisted surgical repair of a congenital lumbar hernia involving the superior lumbar triangle (Grynfeltt-Lesshaft hernia) in a two-year-old female patient.

## Case presentation

A two-year-old female patient, with a history of lumbo-costo-vertebral syndrome (LCVS) and previous bilateral open inguinal hernia repair, was referred to the outpatient pediatric surgery clinic with intermittent left flank bulging associated with crying, coughing, and laughing. There was no associated pain, history of irreducibility, or trauma. On physical examination, the patient had an abnormally prominent right costal margin and scoliosis convex to the left. The hernia was difficult to identify clinically due to the other skeletal abnormalities.

**Figure 1:**
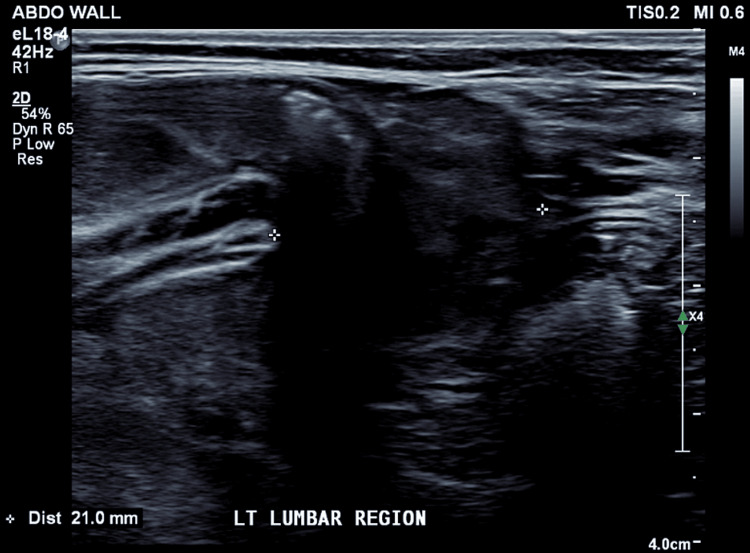
Ultrasound image of the left lumbar region, which demonstrates the hernia containing bowel.

**Figure 2:**
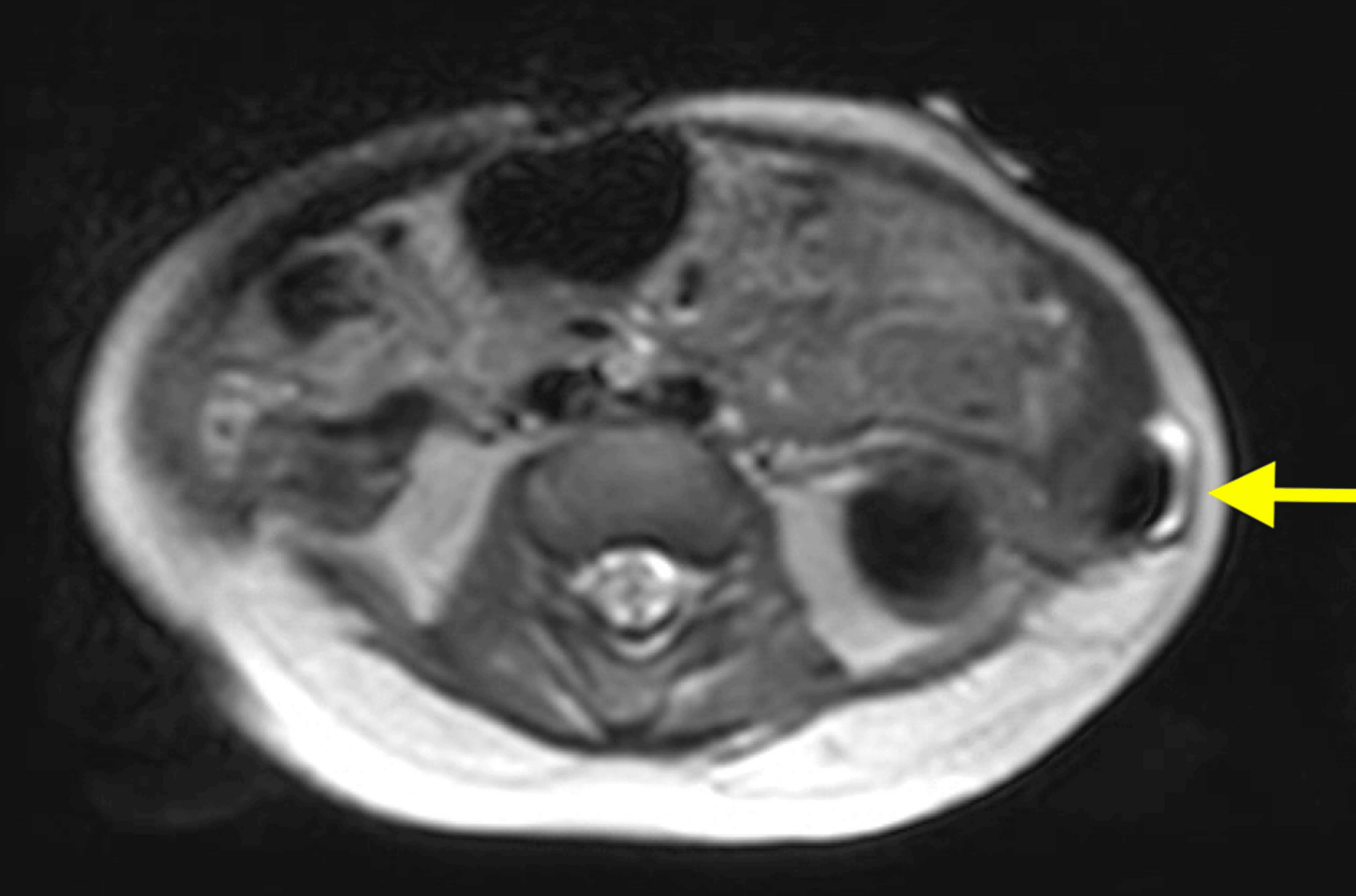
Axial view of T2-weighted MRI image demonstrating the left lumbar hernia containing bowel.

**Figure 3:**
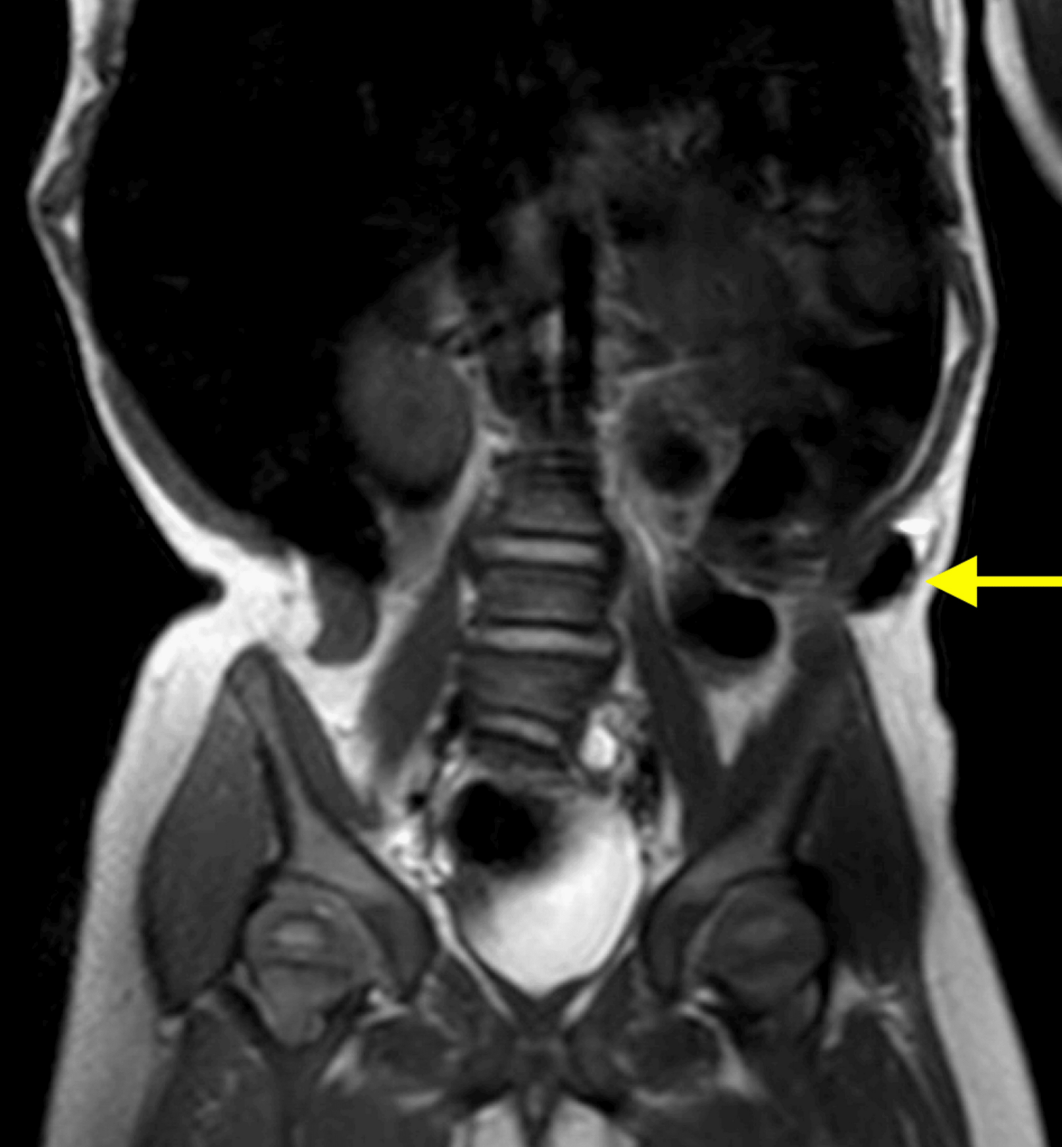
Coronal view of T2-weighted MRI image demonstrating the left lumbar hernia containing bowel.

Chest X-ray showed 10 bilateral rib-bearing thoracic vertebrae, with T11 bearing a left rib. The right ninth and 10th ribs were thickened and mildly dysplastic. Ultrasound assessment revealed a 13x12 mm left lumbar hernia containing a reducible small bowel (Figure 1). Magnetic resonance imaging (MRI) was performed to further characterize the hernia and confirmed an 11 mm left lumbar hernia (Figures 2-3). A hypoplastic S1 vertebrae was noted, with reduced vertebral height on the left, while other abnormalities included a dilated sacral spinal canal, lateral sacral curvature deformity, and left deviation of the natal cleft.

**Figure 4:**
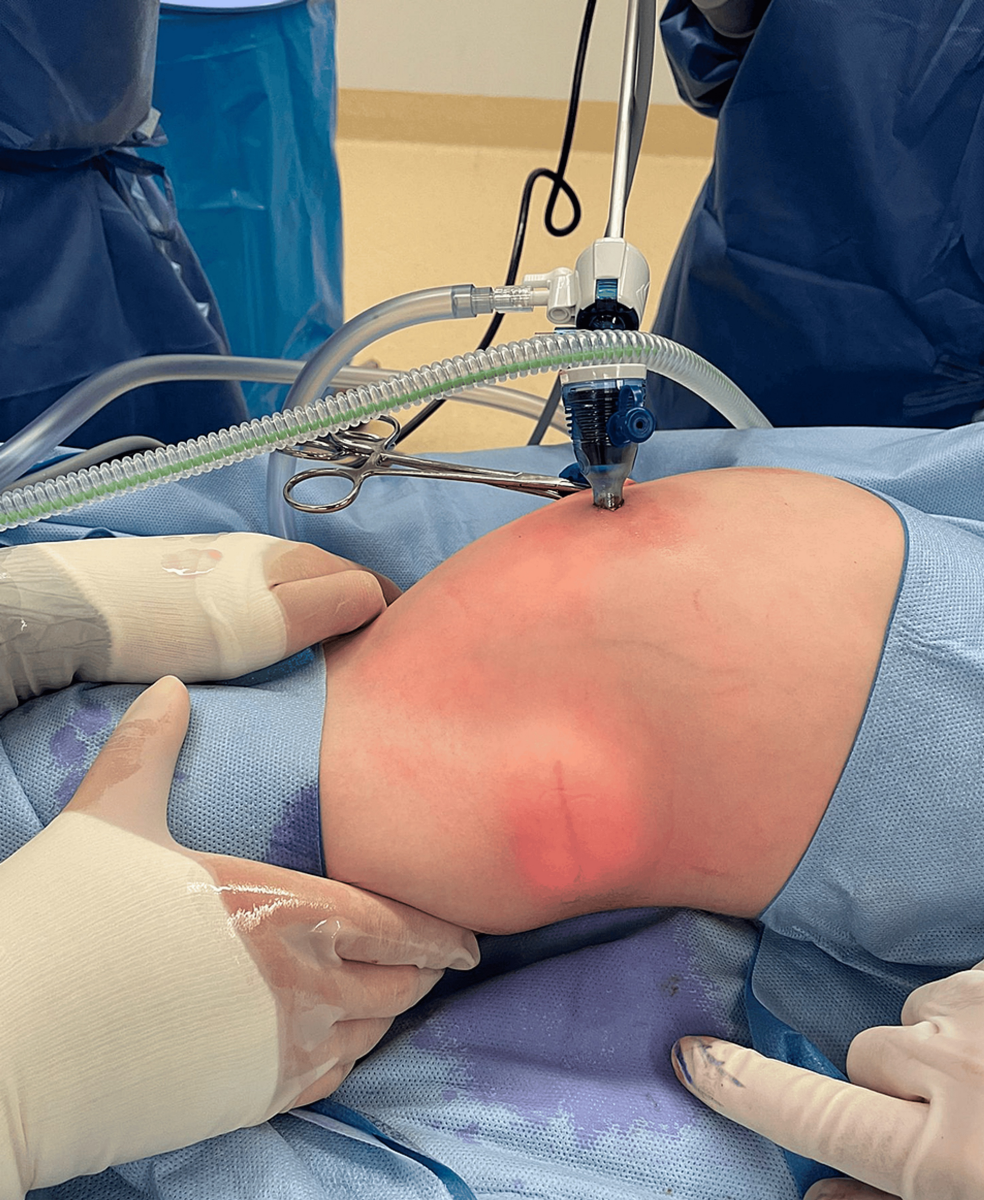
External appearance of the left lumbar hernia after establishing pneumoperitoneum.

**Figure 5:**
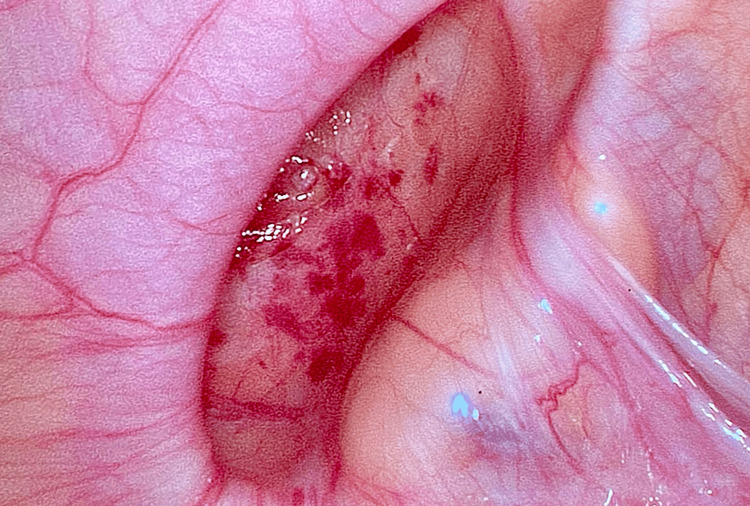
Laparoscopic view of the empty hernia sac.

Given the difficulty of identifying the hernia on clinical examination, the patient was booked for an elective laparoscopic-assisted repair of the lumbar hernia. A 5 mm port was inserted through an infraumbilical incision, and following insufflation, the hernia was located externally (Figure 4) and laparoscopically (Figure 5). A skin incision was made over the hernia, and the muscular defect was closed under direct vision with 2-0 Vicryl sutures. Recovery was uneventful, and she was discharged on day one following the operation. On follow-up review at 15 months post-operatively, the patient was pain-free with no symptoms or signs of recurrence.

## Discussion

Congenital lumbar hernias are exceptionally rare, accounting for 20% of all lumbar hernias. The remaining 80% of lumbar hernias are acquired hernias, typically in the adult population. Causes of acquired hernias include aged-related degeneration, emaciated body habitus, chronic bronchitis, and blunt trauma [3]. Congenital causes of lumbar hernias are thought to arise from embryologic muscular developmental defects. However, other infrequent causes include localized neuropraxia from paravertebral tumors, nerve entrapment in spina bifida, or abdominal masses causing increased intra-abdominal pressure [3]. Pooled analysis and review of 85 congenital lumbar hernia cases found that 72% of patients also had concomitant anatomical and congenital malformations of the spine, and of these, slightly more than half were associated with LCVS. LCVS is theorized to be precipitated by a single somatic defect, possibly due to transient anoxia in early gestation, resulting in one or more abnormalities of the vertebral bodies, ribs, and abdominal musculature. Other associated abnormalities include right-sided renal agenesis, congenital inguinal hernias, malrotation of the gut, anorectal malformations, diaphragmatic eventration, scoliosis, cardiac abnormalities, and neural tube defects [4]. The distorted anatomy caused by these congenital abnormalities and comorbidities can complicate pre-surgical planning.

Anatomically, lumbar hernias can arise from two triangles. The superior lumbar (Grynfeltt-Lesshaft) triangle resembles an upside-down triangle with its base formed by the 12th rib and bounded laterally by the posterior border of the internal oblique muscle and medially by the anterior border of quadratus lumborum. The latissimus dorsi muscle forms the roof, and the floor is formed by the aponeurosis of the transversus abdominis muscle. The inferior lumbar (Petit’s) triangle is an upright triangle bordered by the iliac crest inferiorly, laterally by the posterior extent of the external oblique muscle, and medially by the latissimus dorsi muscle. Its roof is formed by superficial fascia, whereas the floor is formed by the internal oblique muscle. A greater number of congenital lumbar hernias are found to occur in the superior triangle compared to the inferior triangle (42% vs 33%), and 25% of these hernias were further categorized as the ‘diffuse’ subtype that is not limited to either triangle due to congenital muscular defects or aplasia of the 12th rib [5].

Definitive treatment of congenital lumbar hernias involves surgery, where primary repair can be completed by an open, laparoscopic-assisted, or fully laparoscopic approach. Open repair has been most widely reported, with successful repairs described in two separate case series. One series by Sharma et al. reported 16 cases with a median age of three months and median defect size of 6 cm and mesh reserved for defects greater than 10 cm [6]. Rattan et al. subsequently reported 14 cases, all of whom had LCVS and mesh was only used in one larger 7 cm defect [7]. Both series reported a low rate of complications, with a total of three wound infections and no recurrences noted during the follow-up period. Sharma et al.'s [6] study reported a median follow-up duration of three years, whereas Rattan et al. [7] did not report the follow-up duration; hence, the rate of recurrence is unknown. There is no consensus on the defect size to which mesh should be applied. It has been broadly reported that primary repair is recommended in defects less than 5 cm and mesh repair in defects larger than 10 cm. Between 5 and 10 cm, the application of mesh should be considered depending on the circumstances, such as the quality of the abdominal wall musculature or surgeon preference [5].

Laparoscopic repair of a lumbar hernia was first conducted successfully in adult patients through a laparoscopic transabdominal approach by Heniford et al., imitating familiar techniques used in ventral hernia repair and utilizing intraperitoneal mesh [8]. Laparoscopic repair through the extraperitoneal approach is far less common. However, successful repair in an adult patient was subsequently reported by Claus et al., and this technique is theorized to confer improved pain outcomes as the mesh is held in position by intra-abdominal pressure requiring minimal fixation [9]. Laparoscopic techniques are relatively novel and clinical experience is only limited to a handful of case reports regarding clinical application in pediatric surgery. A hybrid laparoscopic-assisted approach, as employed in our case, was helpful with locating the hernia intra-operatively, as it was not clinically evident on physical examination alone. Other advantages included defining the muscular defect, guiding safe open surgery by confirming a complete reduction of hernia contents, and excluding lumbar hernias of the contralateral side. There are very few reported attempts at laparoscopic repair of congenital lumbar hernias in infants and children. Jones et al. were the first to report safe and successful direct laparoscopic suture repair in a 31-month-old child with LCVS [1]. Following this, Zwaveling et al. [2] documented a case involving an infant who underwent laparoscopic primary suture repair of three bilateral congenital lumbar hernias; this approach advantageously granted access to hernias on both sides. This patient developed recurrences in all three sites initially but eventually had successful definitive repair after the third laparoscopic revisional operation within six months, with the utilization of a mesh [2]. The proposed advantages of a laparoscopic procedure include reduced postoperative pain, shorter length of hospitalization, quicker return to normal daily activities, and an improved cosmetic outcome [10,11].

## Conclusions

A congenital lumbar hernia is a rare type of hernia, particularly affecting children with LCVS. Associated abnormalities of the ribs, vertebral body, and abdominal musculature increase surgical and anesthetic complexities when planning an operation. This case report is the first to describe a hybrid laparoscopic-assisted approach in a pediatric congenital lumbar hernia repair, which enabled precise preoperative localization of the hernia followed by a definitive open surgical repair. Previous studies have focused solely on either open or laparoscopic hernia repair techniques; this hybrid approach offers a new strategy to improve surgical accuracy and safety, particularly in cases where imaging and clinical examination are inconclusive. Our literature review confirms that both open and laparoscopic repairs are safe and feasible, with the choice largely dependent on surgeon preference and expertise. This study provides new evidence supporting a hybrid approach, which may enhance surgical outcomes in complex cases.
